# Supplementary material for: Evaluation of usability and acceptability of a Peruvian telemental health service for early assessment among vulnerable occupational workers: Mixed-method study with a user-centered design approach
Source: PLoS One. 2026 Feb 26;21(2):e0343587. doi: 10.1371/journal.pone.0343587 (PMC12944756; doi:10.1371/journal.pone.0343587)
Supplement: S1 Fig — (DOCX) [file pone.0343587.s001.docx]

Supplementary material 1. Mixed Methods Article Reporting Standards.

| **Section/Topic** | **Item No** | **Checklist Item** | **Page** |
| --- | --- | --- | --- |
| **Title** |  |  |  |
|  | 1a | Quant - Identify the populations studied. | pp2 |
|  | 1b | Mixed - Refrain from using words that are either qualitative (e.g., "explore," "understand") or quantitative (e.g., "determinants," "correlates"), because mixed methods stands in the middle between qualitative and quantitative research. | pp2 |
|  | 1c | Mixed - Reference the mixed methods, qualitative methods, and quantitative methods used. | pp2 |
| **Abstract** |  |  |  |
|  | 2a | Mixed - Indicate the mixed methods design, including types of participants or data sources, analytic strategy, main results/findings, and major implications/significance. | pp2 |
| **Introduction** |  |  |  |
|  | 3a | Quant - State the importance of the problem, including theoretical or practical implications. | pp4-5 |
|  | 3b | Qual - Review, critique, and synthesize the applicable literature to identify key issues/debates/theoretical frameworks in the relevant literature to clarify barriers, knowledge gaps, or practical needs. | pp4-5 |
|  | 3c | Mixed - State three types of research objectives/aims/goals: qualitative, quantitative, and mixed methods. Order these goals to reflect the type of mixed methods design used. | pp4-5 |
| **Method** |  |  |  |
|  | 4a | Mixed -Explain why mixed methods research is appropriate as a methodology given the paper’s goals. | pp6 |
|  | 4b | Mixed - Indicate the qualitative approach to inquiry and the quantitative design used within the mixed methods design type. | Pp7-8 |
|  | 4c | Quant - Report inclusion and exclusion criteria, including any restrictions based on demographic characteristics. | Not appplicable |
|  | 4d | Qual - Describe the participants/data source selection process and inclusion/exclusion criteria. | Not appplicable |
|  | 4e | Quant - Describe settings and locations where data were collected as well as dates of data collection. | pp6 |
|  | 4f | Qual - Provide the general context for the study (when data were collected, sites of data collection). | pp6 |
|  | 4g | Quant - Define all primary and secondary measures and covariates. | Pp9-10 |
|  | 4h | Quant - Report major demographic characteristics and important topic-specific characteristics. | Pp9-10 |
|  | 4i | Quant - Describe procedures for selecting participants. | Pp7 |
|  | 4j | Quant - Describe agreements and payments made to participants. | Pp10 |
|  | 4k | Qual - State the form of data collected (e.g., interviews, questionnaires, media, observation). | Pp10 |
|  | 4l | Qual - Describe questions asked in data collection: content of central questions, form of questions (e.g., open vs. closed). | Pp8-10 |
|  | 4m | Qual - For interview and written studies, indicate the mean and range of the time duration in the data-collection process. | Pp8-10 |
|  | 4n | Qual - Describe any incentives or compensation, and provide assurance of relevant ethical processes of data collection and consent process as relevant. | Pp10 |
|  | 4o | Describe institutional review board agreements, ethical standards met, and safety monitoring. | Pp10 |
| **Results** |  |  |  |
|  | 5a | Quant - Provide information detailing the statistical and data- analytic methods used. | PP14-16 |
|  | 5b | Qual - Describe research findings (e.g., themes, categories, narratives) and the meaning and understandings that the researcher has derived from the data analysis. | Pp10-11 |
|  | 5c | Qual - Demonstrate the analytic process of reaching findings (e.g., quotes, excerpts of data). | Pp10-11 |
|  | 5d | Mixed - Indicate how the qualitative and quantitative results were mixed. | Pp16 |
| **Discussion** |  |  |  |
|  | 6a | Describe the central contributions and their significance in advancing disciplinary understandings. | Pp17-20 |
|  | 6b | Discuss similarities and differences between reported results and work of others. | Pp20-21 |
|  | 6c | Identify the study’s strengths and limitations. | Pp22 |
