# Supplementary figures and images for: Evaluation of usability and acceptability of a Peruvian telemental health service for early assessment among vulnerable occupational workers: Mixed-method study with a user-centered design approach
Source: PLoS One. 2026 Feb 26;21(2):e0343587. doi: 10.1371/journal.pone.0343587 (PMC12944756; doi:10.1371/journal.pone.0343587)

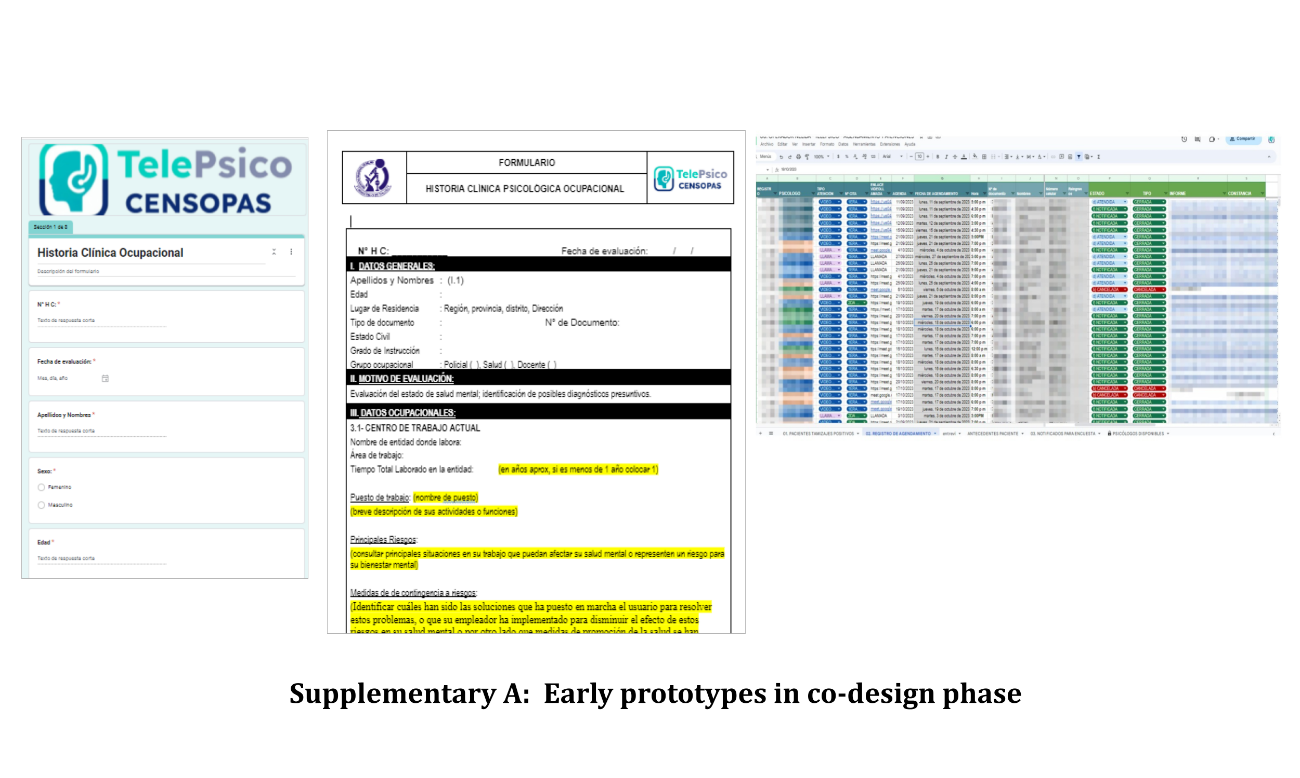


**Supplementary material 2.** Early prototypes in co-design phase.

Supplement: S2 Fig — (DOCX) [file pone.0343587.s002.docx]

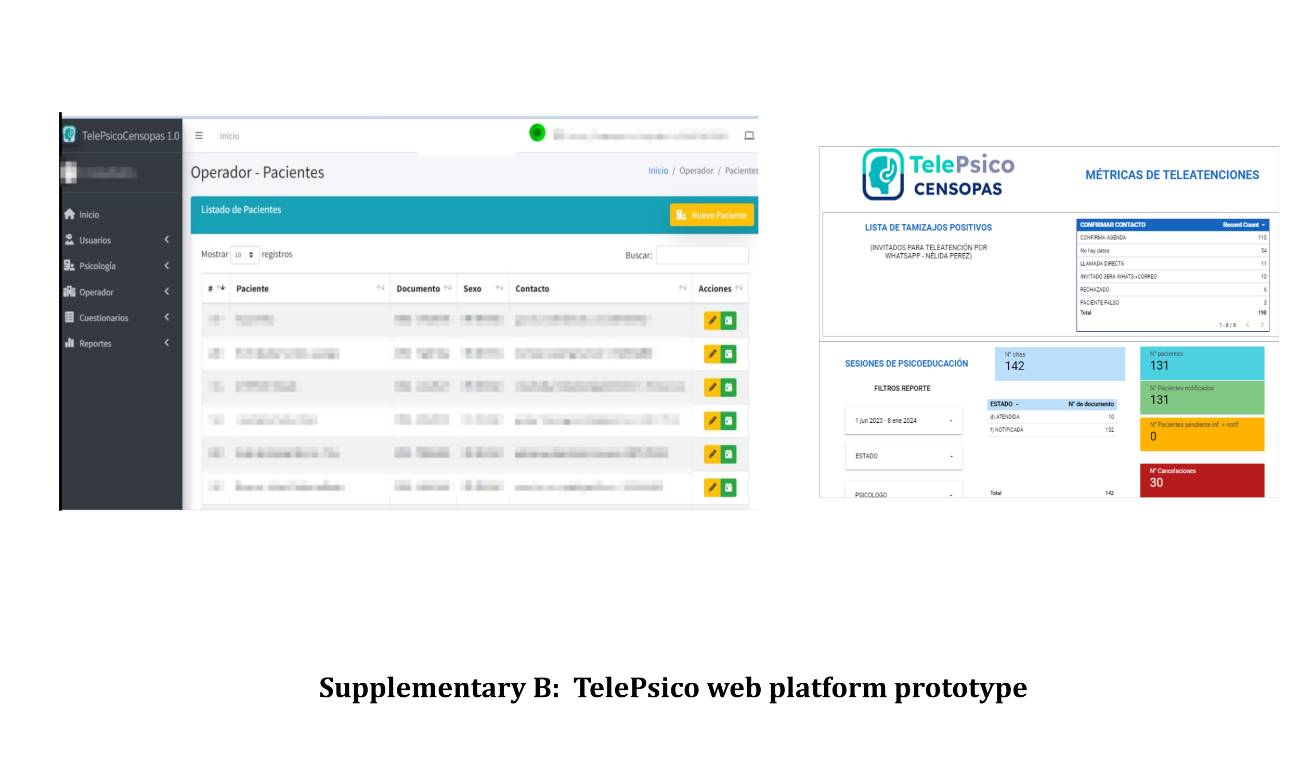


**Supplementary material 3.** TelePsico web platform prototype.

Supplement: S3 Fig — (DOCX) [file pone.0343587.s003.docx]
