# Supplementary material for: Evaluation of usability and acceptability of a Peruvian telemental health service for early assessment among vulnerable occupational workers: Mixed-method study with a user-centered design approach
Source: PLoS One. 2026 Feb 26;21(2):e0343587. doi: 10.1371/journal.pone.0343587 (PMC12944756; doi:10.1371/journal.pone.0343587)
Supplement: S4 Fig — (DOCX) [file pone.0343587.s004.docx]

**Supplementary material 4.** Distribution of workers identified with mental health risk symptoms by job position

| **Job Position** | **Depression** | **Anxiety** | **Post-Traumatic Stress** | **Depression + Anxiety** | **Anxiety + Post-Traumatic Stress** | **Post-Traumatic Stress + Depression** | **Depression, Anxiety, and Post-Traumatic Stress** | **Total** |
| --- | --- | --- | --- | --- | --- | --- | --- | --- |
| **Education Worker** |  |  |  |  |  |  |  |  |
| Teacher | 3 | 0 | 7 | 2 | 0 | 0 | 8 | 20 |
| Education Administrative Staff | 5 | 0 | 8 | 1 | 4 | 2 | 7 | 27 |
|  |  |  |  |  |  |  |  |  |
| **Police Workers** |  |  |  |  |  |  |  |  |
| Police Officers | 0 | 1 | 1 | 0 | 0 | 0 | 1 | 3 |
| Police Administrative Staff | 0 | 0 | 0 | 0 | 0 | 0 | 0 | 0 |
|  |  |  |  |  |  |  |  |  |
| **Healthcare Workers** |  |  |  |  |  |  |  |  |
| Healthcare | 13 | 5 | 30 | 3 | 9 | 7 | 31 | 98 |
| Healthcare Administrative Staff | 9 | 1 | 15 | 1 | 2 | 6 | 11 | 45 |
| **Total** | 30 | 7 | 61 | 7 | 15 | 15 | 58 | 193 |
