# Supplementary material for: Evaluation of usability and acceptability of a Peruvian telemental health service for early assessment among vulnerable occupational workers: Mixed-method study with a user-centered design approach
Source: PLoS One. 2026 Feb 26;21(2):e0343587. doi: 10.1371/journal.pone.0343587 (PMC12944756; doi:10.1371/journal.pone.0343587)
Supplement: S5 Fig — (DOCX) [file pone.0343587.s005.docx]

**Supplementary material 5.** Distribution of satisfaction and usability scores in external users (ESCOMA and SUS) according to sociodemographic characteristics.

|  | **Satisfaction with the service**  **Scale of satisfaction of users of outpatient medical consultation (ESCOMA)** | | | | **Usability of the Digital Platform**  **Scale of the Computer System Usability Questionnaire**  **(CSUQ version 3)** | | | | | | | |
| --- | --- | --- | --- | --- | --- | --- | --- | --- | --- | --- | --- | --- |
| **Sociodemographic Variables** | **D1 = Satisfaction with care processes** | | **D3 = Satisfaction with psychological care** | | **D1: System quality** | | **D2: Information quality** | | **D3: Interface quality** | | **Usability Score** | |
|  | Media | DS | Media | DS | Media | DS | Media | DS | Media | DS | Media | DS |
| **Sex** |  |  |  |  |  |  |  |  |  |  |  |  |
| Female | 45.3 | 7.4 | 37.0 | 5.3 | 38.4 | 5.4 | 36.7 | 6.7 | 24.4 | 5.2 | 87.0 | 16.5 |
| Male | 44.1 | 6.4 | 35.5 | 4.6 | 36.1 | 6.3 | 34.4 | 7.8 | 23.9 | 4.4 | 81.6 | 18.8 |
|  |  |  |  |  |  |  |  |  |  |  |  |  |
| **Age Group:** |  |  |  |  |  |  |  |  |  |  |  |  |
| a) <25 years | 46.5 | 4.8 | 37.1 | 4.0 | 40.5 | 2.0 | 39.3 | 3.9 | 27.0 | 1.8 | 94.5 | 7.6 |
| b) 25-35 years | 43.7 | 8.2 | 36.1 | 6.3 | 37.1 | 6.4 | 35.4 | 7.1 | 24.2 | 4.3 | 84.1 | 17.7 |
| c) 36-45 years | 47.4 | 5.4 | 38.0 | 3.2 | 39.6 | 4.8 | 38.2 | 6.4 | 24.8 | 5.5 | 90.1 | 16.3 |
| d) 46-55 years | 43.3 | 10.1 | 35.7 | 6.4 | 36.5 | 2.1 | 31.7 | 8.7 | 20.0 | 8.8 | 75.2 | 17.7 |
| e) 55-65 years | 42.7 | 2.5 | 34.3 | 4.0 | 30.0 | 5.2 | 31.7 | 6.7 | 21.7 | 4.0 | 70.1 | 16.5 |
|  |  |  |  |  |  |  |  |  |  |  |  |  |
| **Job Position:** |  |  |  |  |  |  |  |  |  |  |  |  |
| Education workers (teaching). | 46.8 | 3.3 | 37.7 | 3.0 | 39.2 | 3.5 | 36.2 | 7.1 | 24.7 | 3.3 | 87.5 | 13.9 |
| Administrative education workers. | 45.5 | 10.2 | 36.5 | 6.7 | 37.5 | 7.9 | 35.3 | 9.6 | 25.0 | 5.8 | 85.2 | 24.0 |
| Operational police workers. | 50.0 | 0.0 | 38.5 | 0.7 | 41.5 | 0.7 | 39.0 | 1.4 | 28.0 | 0.0 | 96.4 | 0.7 |
| Administrative health workers. | 46.5 | 5.2 | 38.1 | 2.8 | 39.6 | 2.9 | 39.4 | 3.6 | 26.0 | 3.0 | 92.6 | 9.3 |
| Healthcare workers. | 43.9 | 7.6 | 35.9 | 5.9 | 37.0 | 6.1 | 35.0 | 7.2 | 23.2 | 5.7 | 82.5 | 17.7 |
|  |  |  |  |  |  |  |  |  |  |  |  |  |
| **Work Modality:** |  |  |  |  |  |  |  |  |  |  |  |  |
| Mixed (remote and in-person) | 47.2 | 6.5 | 37.4 | 6.2 | 38.5 | 6.4 | 37.5 | 6.0 | 26.1 | 3.0 | 89.6 | 15.0 |
| In-person | 44.4 | 7.4 | 36.4 | 4.9 | 37.9 | 5.4 | 36.0 | 7.2 | 23.8 | 5.5 | 85.0 | 17.5 |
| Remote | 48.5 | 2.1 | 39.5 | 0.7 | 38.0 | 5.7 | 37.0 | 7.1 | 25.0 | 4.2 | 87.5 | 17.7 |
|  |  |  |  |  |  |  |  |  |  |  |  |  |
| **Have you been diagnosed with any mental health problem?** |  |  |  |  |  |  |  |  |  |  |  |  |
| No | 45.1 | 7.5 | 37.0 | 4.4 | 38.4 | 4.8 | 36.0 | 6.9 | 24.1 | 5.1 | 86.0 | 16.1 |
| Yes, before the pandemic | 44.4 | 7.3 | 35.5 | 7.0 | 36.3 | 8.0 | 36.4 | 7.6 | 24.5 | 5.7 | 84.6 | 21.3 |
| Yes, during the pandemic | 46.5 | 4.8 | 37.5 | 5.9 | 38.6 | 4.0 | 38.3 | 5.4 | 25.9 | 2.7 | 90.4 | 12.3 |
|  |  |  |  |  |  |  |  |  |  |  |  |  |
| **Total** | 45.1 | 7.2 | 36.7 | 5.2 | 38.0 | 5.6 | 36.3 | 6.9 | 24.3 | 5.1 | 86.1 | 16.9 |
