# Supplementary material for: Evaluation of usability and acceptability of a Peruvian telemental health service for early assessment among vulnerable occupational workers: Mixed-method study with a user-centered design approach
Source: PLoS One. 2026 Feb 26;21(2):e0343587. doi: 10.1371/journal.pone.0343587 (PMC12944756; doi:10.1371/journal.pone.0343587)
Supplement: S6 Fig — (DOCX) [file pone.0343587.s006.docx]

**Supplementary material 6:** Distribution of usability rating in external users (SUS) according to sociodemographic characteristics.

|  | **Usability of the Digital Platform**  **Scale of the Computer System Usability Questionnaire (CSUQ version 3)** | | | | | | | | | | | | |
| --- | --- | --- | --- | --- | --- | --- | --- | --- | --- | --- | --- | --- | --- |
| **Sociodemographic Variables** | **a) Best imaginable** | | **b) Excellent** | | **c) Good** | | **d) Ok** | | **e) Poor** | | **f) Horrible** | | **N** |
|  | **n°** | **%** | **n°** | **%** | **n°** | **%** | **n°** | **%** | **n°** | **%** | **n°** | **%** |  |
| **Sex** |  |  |  |  |  |  |  |  |  |  |  |  |  |
|  |  |  |  |  |  |  |  |  |  |  |  |  |  |
| Female | 22 | 32.8% | 23 | 34.3% | 11 | 16.4% | 8 | 11.9% | 1 | 1.5% | 2 | 3.0% | 67 |
| Male | 3 | 21.4% | 3 | 21.4% | 4 | 28.6% | 2 | 14.3% | 2 | 14.3% |  |  | 14 |
|  |  |  |  |  |  |  |  |  |  |  |  |  |  |
| **Age Group** |  |  |  |  |  |  |  |  |  |  |  |  |  |
| a) <25 years | 4 | 36.4% | 5 | 45.5% | 2 | 18.2% |  |  |  |  |  |  | 11 |
| b) 25-35 years | 10 | 27.0% | 12 | 32.4% | 7 | 18.9% | 5 | 13.5% | 2 | 5.4% | 1 | 2.7% | 37 |
| c) 36-45 years | 11 | 45.8% | 7 | 29.2% | 3 | 12.5% | 2 | 8.3% |  |  | 1 | 4.2% | 24 |
| d) 46-55 years |  |  | 2 | 33.3% | 1 | 16.7% | 3 | 50.0% |  |  |  |  | 6 |
| e) 55-65 years |  |  |  |  | 2 | 66.7% |  |  | 1 | 33.3% |  |  | 3 |
|  |  |  |  |  |  |  |  |  |  |  |  |  |  |
| **Job Position** |  |  |  |  |  |  |  |  |  |  |  |  |  |
| Education workers (teaching). | 2 | 33.3% | 2 | 33.3% |  |  | 2 | 33.3% |  |  |  |  | 6 |
| Administrative education workers. | 3 | 27.3% | 5 | 45.5% | 1 | 9.1% |  |  | 1 | 9.1% | 1 | 9.1% | 11 |
| Operational police workers. |  |  | 2 | 100.0% |  |  |  |  |  |  |  |  | 2 |
| Administrative health workers. | 9 | 45.0% | 6 | 30.0% | 4 | 20.0% | 1 | 5.0% |  |  |  |  | 20 |
| Healthcare workers. | 11 | 26.2% | 11 | 26.2% | 10 | 23.8% | 7 | 16.7% | 2 | 4.8% | 1 | 2.4% | 42 |
|  |  |  |  |  |  |  |  |  |  |  |  |  |  |
| **Work Modality** |  |  |  |  |  |  |  |  |  |  |  |  |  |
| Mixed (remote and in-person) | 8 | 44.4% | 4 | 22.2% | 3 | 16.7% | 2 | 11.1% | 1 | 5.6% |  |  | 18 |
| In-person | 16 | 26.2% | 22 | 36.1% | 11 | 18.0% | 8 | 13.1% | 2 | 3.3% | 2 | 3.3% | 61 |
| Remote | 1 | 50.0% |  |  | 1 | 50.0% |  |  |  |  |  |  | 2 |
|  |  |  |  |  |  |  |  |  |  |  |  |  |  |
| **Have you been diagnosed with any mental health problem?** |  |  |  |  |  |  |  |  |  |  |  |  |  |
| No | 16 | 28.6% | 19 | 33.9% | 11 | 19.6% | 7 | 12.5% | 2 | 3.6% | 1 | 1.8% | 56 |
| Yes, before the pandemic | 7 | 41.2% | 3 | 17.6% | 3 | 17.6% | 2 | 11.8% | 1 | 5.9% | 1 | 5.9% | 17 |
| Yes, during the pandemic | 2 | 25.0% | 4 | 50.0% | 1 | 12.5% | 1 | 12.5% |  |  |  |  | 8 |
|  |  |  |  |  |  |  |  |  |  |  |  |  |  |
| **Total** | 25 | 30.9% | 26 | 32.1% | 15 | 18.5% | 10 | 12.3% | 3 | 3.7% | 2 | 2.5% | 81 |
