# Supplementary material for: Evaluation of usability and acceptability of a Peruvian telemental health service for early assessment among vulnerable occupational workers: Mixed-method study with a user-centered design approach
Source: PLoS One. 2026 Feb 26;21(2):e0343587. doi: 10.1371/journal.pone.0343587 (PMC12944756; doi:10.1371/journal.pone.0343587)
Supplement: S7 Fig — (DOCX) [file pone.0343587.s007.docx]

**Supplementary material 7.** User perceptions during usability evaluation through interviews.

| **User Type** | **Topics** | **Percepcions** |
| --- | --- | --- |
| External user | Perceptions about the ease of use of the Platform. | "Easy to use, no need for help."  "Adapted to virtuality, no difficulties." |
|  | Perceptions about understanding the instructions. | "Found the instructions understandable, especially regarding the time to enter the platform."  "Mentioned that everything was fine, although had concerns about the links." |
|  | Perceptions about the element to promote the use of the Platform. | "Emphasize the need to publish relevant information on social networks to reach the community."  "Recommendation to provide detailed instructions, especially for new users on technological platforms."  "Proposal to standardize messages and improve coordination with assigned specialists for more efficient service." |
|  | Suggestions for improving the platform. | "Incorporate a blog or link with information about meeting points, contacts, surveys, and types of available assistance."  "Integrate all functions for a smoother and more personalized experience." |
|  |  |  |
| Usuario interno | Perceptions about ease of use. | "The instructions were understandable, although they consulted during the training to clarify some aspects."  "The last platform, although recently used, seemed manageable and better in terms of organization and stress reduction." |
|  | Perceptions about the attention provided within the platform. | "He believes that the intervention is more suitable for professionals and suggests that for the general public, it should be shorter, more practical, and synthesized."  "He comments on the importance of the accuracy of responses in extensive questionnaires and suggests the need for motivation and availability to complete them." |
|  | Suggestions to promote the use of the platform. | "He proposes that the record and medical history be more concise to effectively address user issues."  "He considers it important to promote the platform and suggests conducting in-person activities, such as workshops, aimed at vulnerable groups." |
|  | Perceptions about training for platform use. | "He highlights the usefulness of practice with practical cases to better understand the platform system."  "He considers the training to be clear and effective for carrying out the care." |
