# Supplementary material for: Evaluation of usability and acceptability of a Peruvian telemental health service for early assessment among vulnerable occupational workers: Mixed-method study with a user-centered design approach
Source: PLoS One. 2026 Feb 26;21(2):e0343587. doi: 10.1371/journal.pone.0343587 (PMC12944756; doi:10.1371/journal.pone.0343587)
Supplement: S8 Fig — (DOCX) [file pone.0343587.s008.docx]

**Supplementary material 8.** Service satisfaction qualification levels by ESCOMA instrument D1 and D3 dimensions

**
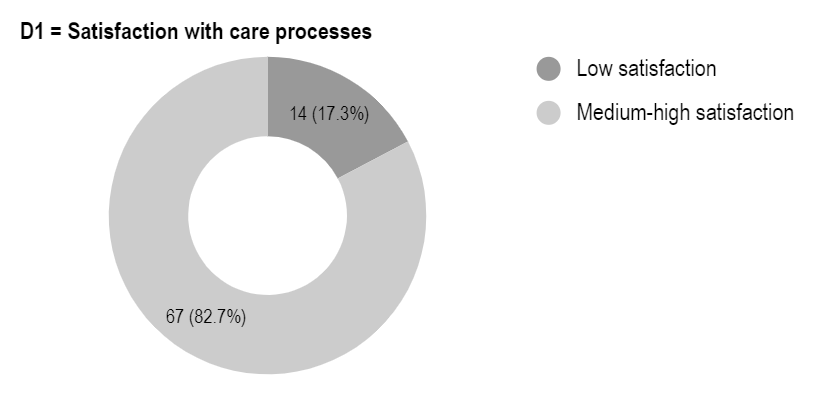
**

**
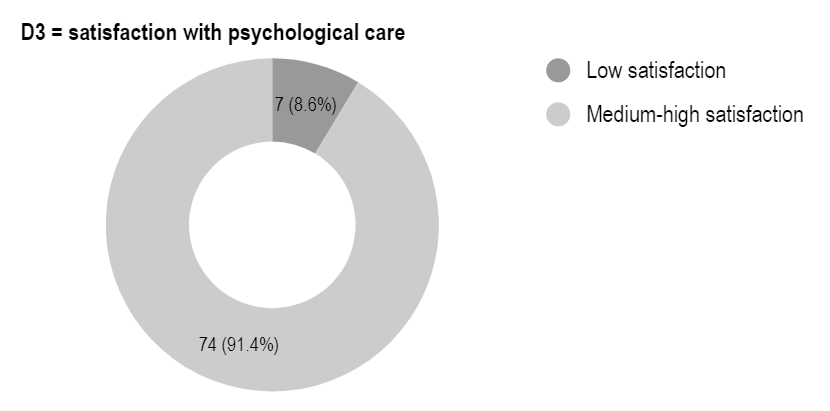
**
