# Supplementary material for: Evaluation of usability and acceptability of a Peruvian telemental health service for early assessment among vulnerable occupational workers: Mixed-method study with a user-centered design approach
Source: PLoS One. 2026 Feb 26;21(2):e0343587. doi: 10.1371/journal.pone.0343587 (PMC12944756; doi:10.1371/journal.pone.0343587)
Supplement: S9 Fig — (DOCX) [file pone.0343587.s009.docx]

**Supplementary material 9.** Perceptions of external and internal users regarding the acceptability of the service.

| **Tipo de usuario** | **Temas** | **Percepciones** |
| --- | --- | --- |
| Usuario externo | Perceptions about the user experience with the telecare platform. | "Good experience, especially for having a sensitivity profile."  "Very good and accessible experience." |
|  | Perceptions of the positive aspects of the Platform. | "No complications found, considered practical for communication with the professional."  "The direct contact part with the specialist and the development of questionnaires were simple and understandable." |
|  | Perception of the negative aspects of the Platform. | "Lack of clarity in the initial presentation via WhatsApp about the purpose of the contact." |
|  | Perception of the experience of adapting to technical requirements. | "Use without significant difficulties from the computer."  "Limited focus on the platform, mainly on filling out questionnaires."  "Lack of explicit invitation to explore other sections of the platform." |
|  | Perceptions of the problem with the use of the Internet. | "No experienced problems with internet connection." |
|  | Perceptions of the problem with the use of technological equipment. | "Confusion experienced with platform links."  "Preference for video conferencing for a more personal interaction." |
|  | Perceptions about the security of the Platform. | "Perceived security when receiving the link in a group of health professionals."  "Trust was supported by the endorsement of institutions and professionals in the talks." |
|  | Sensations about the attention provided within the platform. | "Perceived utility in the care provided, especially in guidance for coping with work-related issues."  "Positive assessment of the experience, highlighting satisfaction and motivation to continue using the service." |
|  | Perceptions about the difficulties for the service to be sustained. | "Recommendation to promote the platform on social networks like Facebook and Instagram."  "Suggestion to improve support response to be more immediate." |
|  |  |  |
| Usuario interno | Perceptions about the experience with the platform. | "Easy adaptation to the new platform." |
|  | Perceptions of the positive aspects. | "Ability to see scheduled users and schedule appointments."  "Fill out the medical history in real-time during the appointment." |
|  | Perceptions of the negative aspects. | "Need for improvements in time optimization, especially with multiple tabs for different functions." |
|  | Perceptions of the future use of the platform. | "Mentions the possibility of difficulties in rural areas due to internet connection interference and lack of knowledge about platforms."  "Highlights the importance of training on the use of technological equipment, such as computers." |
|  | Difficulties for the service to be sustained over time. | "Highlights the need for dissemination and training to overcome barriers, proposing talks and workshops in communities."  "Identifies accessibility as a limitation, especially in towns without internet connection or with limited access to electricity." |
