# Supplementary material for: Evaluation of usability and acceptability of a Peruvian telemental health service for early assessment among vulnerable occupational workers: Mixed-method study with a user-centered design approach
Source: PLoS One. 2026 Feb 26;21(2):e0343587. doi: 10.1371/journal.pone.0343587 (PMC12944756; doi:10.1371/journal.pone.0343587)
Supplement: Attachment — Submitted filename: Response letter.docx [file pone.0343587.s011.docx]

PONE-D-25-40617

Evaluation of usability and acceptability of a Peruvian Telemental health service for early assessment among vulnerable occupational workers: mixed-method study with a user-centered design approach

PLOS One

**Journal Requirements:**

When submitting your revision, we need you to address these additional requirements.

1. Please ensure that your manuscript meets PLOS ONE's style requirements, including those for file naming. The PLOS ONE style templates can be found at https://journals.plos.org/plosone/s/file?id=wjVg/PLOSOne_formatting_sample_main_body.pdf and https://journals.plos.org/plosone/s/file?id=ba62/PLOSOne_formatting_sample_title_authors_affiliations.pdf

**Reply:** We confirm that our manuscript complies with all PLOS ONE style requirements.

2. Your ethics statement should only appear in the Methods section of your manuscript. If your ethics statement is written in any section besides the Methods, please delete it from any other section.

**Reply:** We have added a subsection entitled “Ethical Considerations” to the manuscript, within the Methods section:

*“In general, the study provided online informed consent to all participants, who took part voluntarily. Informed consent was signed virtually by participants by clicking on the “I agree to participate” option. Additionally, the identity of participants in the interviews, workshops, and surveys was protected through data anonymization and the custody of information with controlled access levels. Participants did not receive any type of monetary contribution for their participation.*

*This study was approved by the ethics and research committee of the National Institute of Health of Peru, through Directorial Resolution No. 206-2023-OGITT-INS (COD. OC-048-22).”*

3. We note that the grant information you provided in the ‘Funding Information’ and ‘Financial Disclosure’ sections do not match.
When you resubmit, please ensure that you provide the correct grant numbers for the awards you received for your study in the ‘Funding Information’ section.

**Reply:** We confirm that this information has been updated in the submission and that both sections contain the same information.

4. Thank you for stating the following in the Acknowledgments Section of your manuscript:
We express our gratitude to the Spanish Agency for International Development Cooperation (AECID, in spanish) and the National Program for Scientific Research and Advanced Studies (PROCIENCIA-CONCYTEC-Peru, in spanish), who funded the project, enabling its execution, under contract number 076-2021-PROCIENCIA

We note that you have provided funding information that is not currently declared in your Funding Statement. However, funding information should not appear in the Acknowledgments section or other areas of your manuscript. We will only publish funding information present in the Funding Statement section of the online submission form.
Please remove any funding-related text from the manuscript and let us know how you would like to update your Funding Statement. Currently, your Funding Statement reads as follows:
The author(s) received no specific funding for this work.

Please include your amended statements within your cover letter; we will change the online submission form on your behalf.

**Reply:** We have made the requested modifications:

*“Acknowledgments*

*Not applicable.*

*Funding Information*

*We express our gratitude to the Spanish Agency for International Development Cooperation (AECID, in spanish) and the National Program for Scientific Research and Advanced Studies (PROCIENCIA-CONCYTEC-Peru, in spanish), who funded the project, enabling its execution, under contract number 076-2021-PROCIENCIA.”*

5. Please amend your list of authors on the manuscript to ensure that each author is linked to an affiliation. Authors’ affiliations should reflect the institution where the work was done (if authors moved subsequently, you can also list the new affiliation stating “current affiliation:….” as necessary).

**Reply:** We confirm that the names, affiliations, email addresses, and ORCID identifiers are correct in all cases.

6. Please remove your figures from within your manuscript file, leaving only the individual TIFF/EPS image files, uploaded separately. These will be automatically included in the reviewers’ PDF**.**

**Reply:** The images were included separately in TIFF format.

7. We note that Figure 1, Supplementary A, Supplementary B, Supplementary C, Supplementary D, in your submission contain copyrighted images. All PLOS content is published under the Creative Commons Attribution License (CC BY 4.0), which means that the manuscript, images, and Supporting Information files will be freely available online, and any third party is permitted to access, download, copy, distribute, and use these materials in any way, even commercially, with proper attribution. For more information, see our copyright guidelines: http://journals.plos.org/plosone/s/licenses-and-copyright.

We require you to either (1) present written permission from the copyright holder to publish these figures specifically under the CC BY 4.0 license, or (2) remove the figures from your submission:

a. You may seek permission from the original copyright holder of Figure 1, Supplementary A, Supplementary B, Supplementary C, Supplementary D, to publish the content specifically under the CC BY 4.0 license.

We recommend that you contact the original copyright holder with the Content Permission Form (http://journals.plos.org/plosone/s/file?id=7c09/content-permission-form.pdf) and the following text:
“I request permission for the open-access journal PLOS ONE to publish XXX under the Creative Commons Attribution License (CCAL) CC BY 4.0 (http://creativecommons.org/licenses/by/4.0/). Please be aware that this license allows unrestricted use and distribution, even commercially, by third parties. Please reply and provide explicit written permission to publish XXX under a CC BY license and complete the attached form.”

Please upload the completed Content Permission Form or other proof of granted permissions as an "Other" file with your submission.

In the figure caption of the copyrighted figure, please include the following text: “Reprinted from [ref] under a CC BY license, with permission from [name of publisher], original copyright [original copyright year].”

b. If you are unable to obtain permission from the original copyright holder to publish these figures under the CC BY 4.0 license or if the copyright holder’s requirements are incompatible with the CC BY 4.0 license, please either i) remove the figure or ii) supply a replacement figure that complies with the CC BY 4.0 license. Please check copyright information on all replacement figures and update the figure caption with source information. If applicable, please specify in the figure caption text when a figure is similar but not identical to the original image and is therefore for illustrative purposes only.
**Reply:** The figures and supplementary materials under review correspond to images that are the property of the authors of the present article; therefore, it is not necessary to request licenses from third parties. Likewise, it has been indicated in the “Supporting Information” section that the supplementary files are of original authorship.

8. [Please include captions for your Supporting Information files at the end of your manuscript, and update any in-text citations to match accordingly. Please see our Supporting Information guidelines for more information: http://journals.plos.org/plosone/s/supporting-information.](https://www.editorialmanager.com/pone/Please%20include%20captions%20for%20your%20Supporting%20Information%20files%20at%20the%20end%20of%20your%20manuscript,%20and%20update%20any%20in-text%20citations%20to%20match%20accordingly.%20Please%20see%20our%20Supporting%20Information%20guidelines%20for%20more%20information:%20http:/journals.plos.org/plosone/s/supporting-information.%20)

**Reply:** We have included the captions for the Supporting Information files at the end of our manuscript.

*“Supporting Information*

*Supplementary A. Early prototypes in co-design phase.*

*Supplementary B. TelePsico web platform prototype.*

*Supplementary C. Public information of the service.*

*Supplementary D. Telecare reports.*

*Supplementary material 1. Distribution of workers identified with mental health risk symptoms by job position*

*Supplementary material 2. Distribution of satisfaction and usability scores in external users (ESCOMA and SUS) according to sociodemographic characteristics.*

*Supplementary material 3. Distribution of usability rating in external users (SUS) according to sociodemographic characteristics.*

*Supplementary material 4. User Perceptions during Usability Evaluation through Interviews.*

*Supplementary material 5. Service satisfaction qualification levels by ESCOMA instrument D1 and D3 dimensions*

*Supplementary material 6. Perceptions of external and internal users regarding the acceptability of the service.”*

9. If the reviewer comments include a recommendation to cite specific previously published works, please review and evaluate these publications to determine whether they are relevant and should be cited. There is no requirement to cite these works unless the editor has indicated otherwise.

**Reply:** Ok.

10. Please review your reference list to ensure that it is complete and correct. If you have cited papers that have been retracted, please include the rationale for doing so in the manuscript text, or remove these references and replace them with relevant current references. Any changes to the reference list should be mentioned in the rebuttal letter that accompanies your revised manuscript. If you need to cite a retracted article, indicate the article’s retracted status in the References list and also include a citation and full reference for the retraction notice.
**Reply:** We have reviewed the reference list and confirm that it is correct.

**Review Comments to the Author**

Reviewer #1: Thank you for the opportunity to review this paper. The topic of a national-scale telehealth application for mental health is highly significant. The study is exceptionally well-designed and executed, demonstrating a broad scope and resulting in a practical and currently implemented system. It is commendable that the system is already operational and in use, and I have reviewed the corresponding website.
I have a few specific comments aimed at further strengthening the manuscript:
**Reply:** Thank you for your comments. We will respond to each one.

1- I recommend a thorough check of the manuscript's punctuation and grammar. For instance, in the Abstract (Background section), I noted a sentence concluding with two periods (..) instead of a single one. A general review for consistency in punctuation (commas, periods, colons, etc.) across the entire text is advised.
**Reply:** Thank you for your recommendation. We will respond to each one.

2- In the Introduction section, while the third paragraph makes an essential reference to prevalence data, the Introduction would be significantly improved by explicitly stating the global prevalence of mental health disorders, followed by specific prevalence statistics within Peru. The current text implies the source of the data through citations, but it should explicitly state in the text that these statistics pertain to Peru.
**Reply:** As global prevalence data are already presented in the first paragraph of the Introduction, we have revised the third paragraph to explicitly state that the reported prevalence figures correspond to the Peruvian context.

3- In the Method section, the description of the setting for the system's implementation is currently ambiguous. If the scope is indeed national (national scale), this should be stated explicitly and specifically in the Methods section to reflect the significant scale of the project.
**Reply:** We have revised the Methods section to explicitly clarify that the digital mental health system was implemented at a metropolitan scale, specifically in Lima and Callao.

4- In the Result section, the inclusion of a flowchart (Figure 2) and a view of the initial website page in the Appendix is helpful. However, to better illustrate the user experience and the functional processes outlined in Figure 2 (from screening to the final report), it is recommended to include additional screenshots of the live system/website in the main body of the paper.
**Reply:** Thank you for your recommendation. We will respond to each one.

5- The Discussion is well-written, thoroughly analyzing the work from various angles. To provide richer context and academic rigor, it is suggested to integrate relevant similar studies in the appropriate sections and include a comparative analysis of this work against those studies.
**Reply:** We have revised the Discussion section to more explicitly integrate relevant comparable studies and to include a clearer comparative analysis between our findings and previously published telehealth interventions.

6- If this information is not planned for a separate publication, it would be beneficial to include some current key utilization metrics for the operational system. This could consist of the total number of individuals who have received assistance from the system to date, or other relevant indicators of current scale and impact.

**Reply:** At the time of manuscript submission, key utilization indicators of the TelePsico CENSOPAS system—specifically the number of workers evaluated and sensitized—were available and have already been reported in the Results section under Telehealth service intervention outcomes.

Reviewer #2: This manuscript addresses a timely and significant issue regarding mental health support for vulnerable occupational groups during the COVID-19 pandemic. The application of a User-Centered Design (UCD) framework is a major strength of this study, providing a robust model for developing effective health IT solutions. However, there are specific methodological clarifications and minor corrections required before publication.
**Reply:** Thank you for your comments. We will respond to each one.

1) Methodological Terminology:In the "Usability, satisfaction and acceptability assessment" section, the analysis is described as a "phenomenological approach." Given that the results focus on usability feedback and satisfaction rather than the deep essence of lived experiences, this term seems misapplied. The analysis appears to align more closely with "Thematic Analysis" or "Qualitative Content Analysis." Please revise this terminology to accurately reflect the method used.
**Reply:** We thank the reviewer for this valuable comment. After extensive discussion among the authors, we agree that the reviewer’s comment is correct. Our study focuses on usability- and satisfaction-related comments; therefore, it is better aligned with a thematic analysis. We have revised the text throughout the manuscript to specify that the analysis is thematic.

2) Citation Accuracy: Reference [45] (Universitat Oberta de Catalunya, Interview Toolkit) is currently cited in the context of data analysis using Atlas Ti. This reference pertains to data collection (interviews), not analysis. Please correct this citation to a source relevant to the analytical method employed.
**Reply:** Thank you for your comments. We will respond to each one.

3) Abstract Clarity: The Abstract mentions "81 participants in the efficacy assessment" without sufficient context. To avoid confusion regarding the response rate, please clarify that these 81 participants are a subset of the 134 users who received psychoeducation.
**Reply:** The Abstract was revised in accordance with the reviewer’s suggestion to improve clarity.

4) Comparative Analysis Context: The study compares satisfaction scores with historical outpatient data (Moscoso et al.). While valid as a benchmark, please explicitly acknowledge in the Discussion or Limitations section that comparing a telehealth service (during a pandemic) with traditional outpatient services (pre-pandemic) involves different contexts and user expectations.
**Reply:** We have made the requested modifications:

*“The level of service usage satisfaction, evaluated using the ESCOMA instrument, showed a high score in satisfaction with care processes and psychological care. The evaluation instrument was initially developed by Moscoso et al., who found lower scores in satisfaction levels among users of MINSA, EsSalud, the Armed Forces, and private entities who received outpatient medical care services. These differences could be attributed to system characteristics, such as the freedom of scheduling appointments and the accessibility described earlier, which meet users' needs and expectations [53]. However, it should be noted that the study by Moscoso et al. was conducted at the national level in in-person settings before the COVID-19 pandemic, whereas our study was conducted in telehealth settings during the COVID-19 pandemic. Therefore, the studies may not be directly comparable. Despite this, we consider it a reference proxy that may suggest the high level of satisfaction with the service presented in this study.”*

5) Minor Errors and Typos: Please correct the following errors:
- Abstract: Correct the double period in "complex cases. . Telehealth".
- Abstract-Conclusion: Add a space in "groupsAdditionally".
- Ensure consistent spacing between citations and text throughout the manuscript.

**Reply:** We thank the reviewer for the suggestions. We confirm that we have reviewed the entire text for spelling and writing errors and have corrected them.
